# Activation and Delivery of Tetrazine-Responsive Bioorthogonal Prodrugs

**DOI:** 10.3390/molecules25235640

**Published:** 2020-11-30

**Authors:** Yayue Wang, Chang Zhang, Haoxing Wu, Ping Feng

**Affiliations:** 1 Huaxi MR Research Center, Department of Nuclear Medicine, Frontiers Science Center for Disease-Related Molecular Network, West China Hospital, Sichuan University, Chengdu 610041, China; wangyayue@126.com (Y.W.); chang.zhang1@ucdconnect.ie (C.Z.); 2 Institute of Clinical Trials, West China Hospital, Sichuan University, Chengdu 610041, China

**Keywords:** tetrazine, bioorthogonal reaction, prodrug activation

## Abstract

Prodrugs, which remain inert until they are activated under appropriate conditions at the target site, have emerged as an attractive alternative to drugs that lack selectivity and show off-target effects. Prodrugs have traditionally been activated by enzymes, pH or other trigger factors associated with the disease. In recent years, bioorthogonal chemistry has allowed the creation of prodrugs that can be chemically activated with spatio-temporal precision. In particular, tetrazine-responsive bioorthogonal reactions can rapidly activate prodrugs with excellent biocompatibility. This review summarized the recent development of tetrazine bioorthogonal cleavage reaction and great promise for prodrug systems.

## 1. Introduction

**Figure 1:**
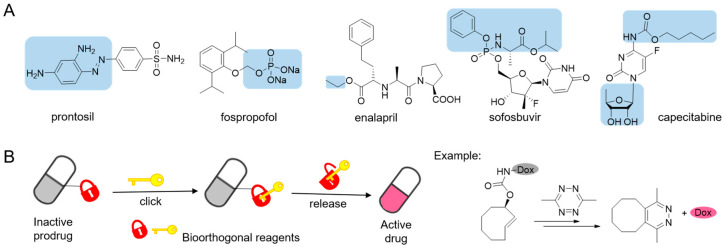
(**A**) Structures of some examples of prodrug (pro-moiety in blue box). (**B**) The concept of prodrug activation based on tetrazine-responsive bioorthogonal click-to-release reaction with representative tetrazine triggered release of doxorubicin (Dox) from *trans*-cyclooctene.

The efficacy of a pharmacotherapy usually depends on whether the drug would achieve its optimal concentration at the specific lesions. Poor physiochemical, biopharmaceutical or pharmacokinetic properties restrict many pharmacologically active compounds from clinical practice [1,2]. One solution to overcome is to administer the drug in a “prodrug” form that remains inert in vitro and converts into its active form in vivo. Prodrugs can provide possibilities to optimize the absorption, distribution, metabolism, excretion and toxicity (ADMET) of the respective parent drugs [3,4]. Prodrug principles as described above, first proposed by Albert in 1958 [5], have been effectively applied to drug design and development [1,6,7,8]. A classic example is prontosil, an antimicrobial sulfanilamide prodrug that ushered the research and development of antimicrobial agents to a new area [9,10]. In recent years, a growing number of prodrugs have been approved in clinical use [1]. Prodrugs are mainly designed to improve the bioavailability of parent drugs for oral delivery and parenteral administration [1,3,4] by increasing aqueous solubility or membrane permeability, for example, anesthetic fospropofol [11], angiotensin-converting enzyme (ACE) inhibitors enalapril [12], and sofosbuvir for hepatitis C virus infection therapy [13] (Figure 1A). On the other hand, prodrugs are playing increasingly important roles in targeted therapy due to either site-directed delivery or site-specific bioactivation [14,15,16,17]. Taking capecitabine as an example (Figure 1A), it undergoes three enzymatic conversions into active antimetabolite drug 5-fluorouracil in tumor tissue [18].

Conventional prodrug activation strategies usually rely on intrinsic physiological changes at the target site such as overexpressed enzymes [19,20], unique microenvironment with lower pH value [21,22], and higher level of reactive oxygen species (ROS) [23,24] and glutathione (GSH) [25,26]. However, these endogenous changes may be too subtle to activate prodrug with high selectivity, resulting in off-target activation that can give rise to adverse effects [27,28]. An alternative is to activate the prodrug with exogenous stimulus such as light [29,30] or robust chemical reactions [2,31]. The latter may be more promising because they are associated with lower cytotoxicity and greater tunability than light-based activation. In addition, light usually cannot reach beyond skin to deeper tissues [32].

Ideal chemical reactant pairs should be stable and avirulent under physiological conditions. They should react exclusively with each other, but not interact with biological functionalities [33]. Bioorthogonal reactions can be considered as good examples of ideal properties mentioned above, and have been widely used in biomedical research [34,35,36,37,38]. The inverse electron demand Diels-Alder (IEDDA) reactions between 1,2,4,5-tetrazine (Tz) and various dienophiles stand out from bioorthogonal reactions for in vivo applications due to the superfast kinetics and excellent biocompatibility [39,40,41]. The tetrazine-responsive bioorthogonal click-to-release reactions result in the cleavage of carbamates, esters and ethers [31], thus providing an opportunity to release functional groups for selective activation of proteins [42,43], fluorogenic probes [44,45] and prodrugs [2,27,46] (Figure 1B) in living systems. These strategies about tetrazine-responsive prodrug activation mainly focus on the cytotoxic chemotherapeutics which usually associated with narrow therapeutic window and severe systemic adverse effects. One of the two bioorthogonal reactants acts as a pro-moiety in the prodrug, and the partner acting as a trigger releases the active drug upon reaction. Only in condition of both reactants coming into proximity will the prodrug be activated. And with a combination of targeted drug delivery systems, the inactive prodrugs can be selectively released without causing toxicity to normal, healthy tissue. Lately, the FDA has approved a first-in-human Phase I clinical trial based on a Click Activated Prodrugs Against Cancer (CAPAC) platform [47]. In this review, we summarized several prodrug-activation chemistries based on tetrazine bioorthogonal cleavage reaction and their further applications for in vivo drug delivery.

## 2. Prodrug-Activation Chemistries

Classical IEDDA bioorthogonal reactions focus on the formation of chemical bonds which lead to stable ligation products. Tetrazines are prone to react with several dienophiles including *trans*-cyclooctenes (TCO) [48,49], norbornene (NB) [50], bicyclononyne (BCN) [51,52,53] and cyclopropane [54,55]. With appropriate structural modifications, an elimination or rearrangement step is allowed to occur after the cycloaddition, releasing a fluorophore, protein residue or active drug with various functional groups, including amino, hydroxyl and carboxyl. In this section we discussed the chemistry characteristics of the emerging tetrazine-responsive bioorthogonal cleavage reactions and their potential for prodrug activation applications.

### 2.1. Tetrazine-Triggered Cleavage from Trans-Cyclooctene

**Figure 2:**
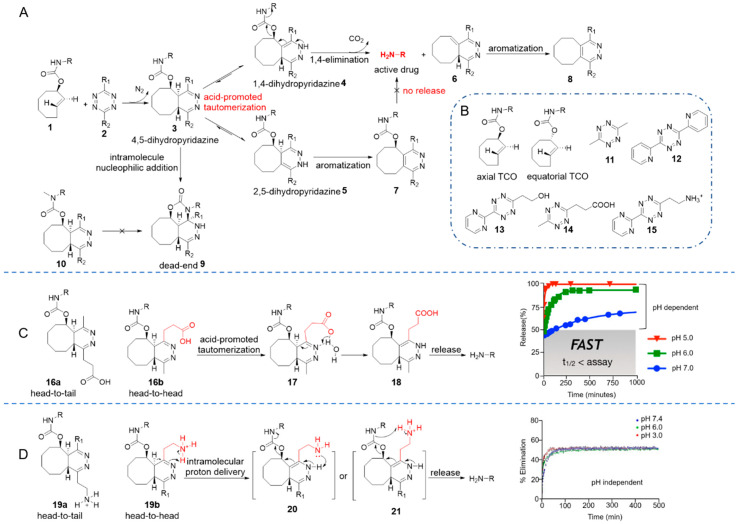
(**A**) Reaction mechanism between cleavable TCO and tetrazines. (**B**) TCO isomers and tetrazines toolbox. (**C**) The mechanism for pH-dependent release of active amine and release profile of **14** at pH 5–7. Reproduced with permission from [58]; © 2020 American Chemical Society. (**D**) The mechanism for pH-independent release of active amine and release profile of **15** at pH 3–7.4. Reproduced with permission from [60]; © 2020 Wiley-VCH Verlag GmbH & Co. KGaA, Weinheim.

In 2013, Robillard and co-workers developed the first bioorthogonal, rapid elimination reaction between tetrazine and *trans*-cyclooctenes (TCOs) [56]. They incorporated a carbamate at the allylic position, resulting in the cleavable TCO **1**. IEDDA cycloaddition between TCO **1** and tetrazine eliminated N_2_ to generate a 4,5-dihydropyridazine intermediate **3**, which tautomerized to 1,4- and 2,5-dihydropyridazine in an aqueous environment. The 1,4-dihydropyridazine **4** spontaneously underwent bond rearrangement to eliminate CO_2_ and an amino-substituted drug (Figure 2A). This hypothesis was confirmed by further experiments [57,58]. They concluded that TCO derivatives with axial carbamates reacted 156-fold faster than the equatorial isomers (Figure 2B) [56,59]. Electron density of the tetrazine was also found to influence the reaction kinetics and the yields of products. For example, dipyridyl-tetrazine **12** reacted rapidly with axial TCO derivates (*k*_2_ = 57.70 M^−1^ s^−1^) but gave a release yield of only 7%. Conversely, dimethyl-tetrazine **11** reacted more slowly (*k*_2_ = 0.54 M^−1^ s^−1^) but gave a higher release yield of 79% [56].

Since substituents on the tetrazine affected reaction kinetics and release yields, Chen and co-workers systematically studied the structure-activity relationship of tetrazine derivatives [61]. Smaller alkyl substituents (such as methyl) led to higher release yield, while a bulky *tert*-butyl group contributed to almost no release. On the other hand, electron-withdrawing groups on the tetrazine promoted cycloaddition while inhibiting elimination. The authors concluded that the reaction would be faster along with higher release yield if unsymmetric tetrazines bearing an electron-withdrawing group at the 3-position and a small, flexible alkyl group at the 6-position were used. For example, pyrimidine-tetrazine **13** allowed over 80% of the fluorescence to recover upon reaction with TCO-coumarin within 30 min, whereas tetrazine **11** only restored 20% of the fluorescence in the same time interval [61].

Further mechanistic studies showed that 1,4-dihydropyridazine **4**, as the key intermediate, underwent an electron-cascade elimination to release the payload, such as a drug. Its rate of formation therefore becomes a limiting factor for the elimination reaction. The 2,5-dihydropyridazine tautomer **5** was slowly oxidized to pyridazine **7** instead of releasing the payload, which limited the overall elimination yield (below 40%) [57]. A detailed investigation of the reaction mechanistic features carried out by the Weissleder group showed that for unsymmetric tetrazines, cycloadditions took place randomly in two orientations (head-to-tail or head-to-head), resulting in different adducts (Figure 2C). Head-to-head adduct **16b** drove the formation of the releasing tautomer **18** and led to fast release. Head-to-tail adduct **16a**, in contrast, contributed to the formation of 2,5-dihydropyridazine, which slowly re-tautomerized to the 1,4-dihydropyridazine tautomer, thereby contributing to release [58].

Acidic conditions accelerate tautomerization and therefore lead to faster and higher release [57,58]. Weissleder and co-workers therefore prepared a plate of acid-functionalized tetrazines and found that the closer the solution pH was to the pK_a_ of the acid, the faster and more complete the release would be. For example, since general acid catalysis facilitates tautomerization, tetrazines **14** bearing a propanoic acid group (pK_a_ = 4.45) were able to release cargos rapidly and with near-quantitative yield at pH 5 (Figure 2C) [58]. However, an unexpected intramolecular cyclization dead-end product **9** was found (Figure 2A). This tricyclic by-product was stable and was slowly oxidized rather than release. But its formation can be blocked by replacing the NH of carbamate with a N-Me group (compound **10**) to enable complete release [58]. In an alternative approach, an aminoethyl moiety was decorated resulting in a series of tetrazines that maintained fast elimination kinetics over a broad pH range from 3.5 to 7.5 [60]. The amine on the aminoethyl group existed as cationic ammonium over the whole biologically relevant pH and acted as an intramolecular catalyst of tautomerization (Figure 2D). Containing an electron-withdrawing group simultaneously, tetrazine **15** promoted elimination at a rate constant to 120 × 10^−5^ s^−1^ without solvent pH dependence [60].

**Figure 3:**
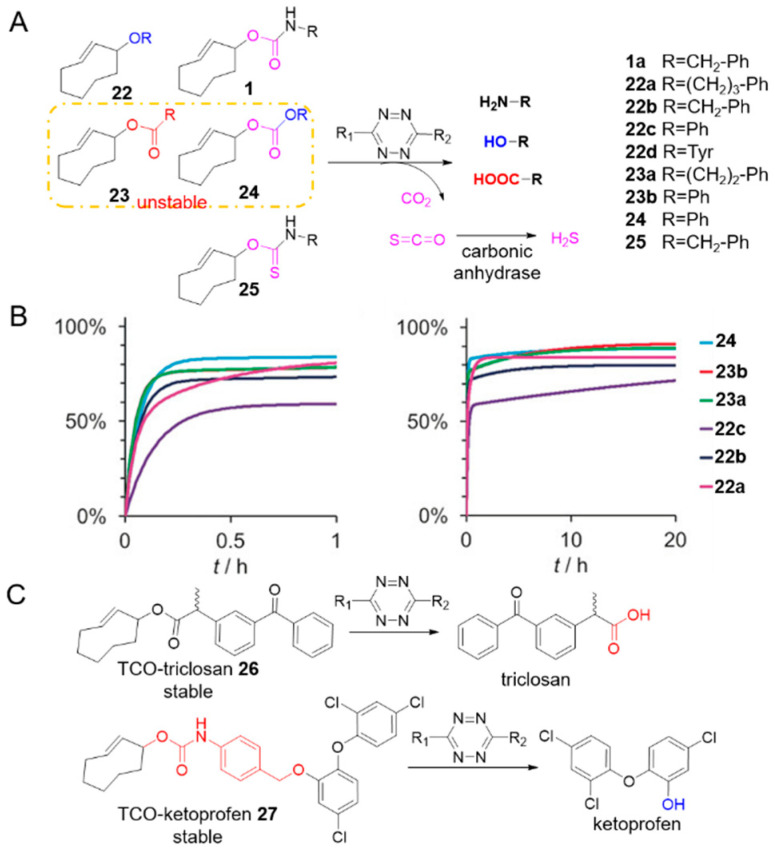
(**A**) The scope of the click-to-release reaction between tetrazine and TCO derivatives. (**B**) Biphasic release from TCO derivates **22**–**25**. Scale: 0–1 h and 0–20 h. Reproduced with permission from [57]; © 2020 Wiley-VCH Verlag GmbH & Co. KGaA, Weinheim (**C**) Stable TCO self-immolative linker and easter prodrug models.

Reactions to unmask other functional groups have also been developed (Figure 3A). Pluth et al. installed a thiocarbamate on TCO (**25**) which reacted with tetrazines to generate carbonyl sulfide/hydrogen sulfide (Figure 3A) [62]. The Robillard group demonstrated the TCOs containing a range of axial allylic substituents including aliphatic, benzylic, and aromatic ethers **22a**–**c**, aromatic and aliphatic esters **23a**, **b** and aromatic carbonate **24**, could react with tetrazine to release the corresponding alcohols, phenols and carboxylic acids (Figure 3A, entries 2–4 in Table 1) [57]. Cycloaddition consumed these TCOs within minutes, and the release exhibited biphasic kinetics profiles with a fast release up to approximately 70–85% within 10–30 min, followed by slower re-tautomerization of 2,5-dihydropyridazine to release the rest within 20 h (Figure 3B) [57]. Despite the broad scope of these reactions, not all chemical groups are useful for in vivo applications: ester bonds (compound **23**) and carbonate (compound **24**), for example, are unstable in plasma and cell medium [57,63,64]. The ether bond (compound **22**), however, is relatively stable in physiological condition: after reacting with tetrazine, TCO caged tyrosine **22d** released the free amino acid for controllable cell growth as a proof of concept verification [57].

To realize the release of alcohols and carboxylic acids, Bernardes’s group applied their insights to the development of stable cleavable predecessors. In 2019, they reported the biocompatible release of alcohols from a TCO-carbamate conjugated with a self-immolative benzyl ether linker (Figure 3C) [63]. The amine was released from the TCO-carbamate, followed by cascaded 1,6-elimination releasing the alcohol. The linker was highly stable in cell culture media and plasma with no release product detected over 15 h and reacted rapidly with tetrazine: cycloaddition was completed within seconds, and half-life of release was approximately 30 min. Activating the OH-containing prodrug of the antibacterial triclosan efficiently killed *E. coli*, although much higher concentrations were needed than triclosan alone (IC_50_ = 298 ± 20 nM vs. 122 ± 10 nM). This lower activity was due to the low release yield of 39%. They also built a more stable TCO ester prodrug model for the release of carboxylic acids (Figure 3C) [64]. The need for bulky substituents to stabilize the ester bond was emphasized. A stable TCO-ketoprofen prodrug **27** was synthesized, and activated rapidly by tetrazine, inhibiting the inflammatory response in living macrophages.

This novel bioorthogonal elimination opens up new avenue for cleavable antibody-drug conjugates and precisely controlled prodrug activation and delivery [2,28,44,72]. But rapid reaction kinetics is a key consideration for implementation in vivo regarding the possible degradation or inactivation of reaction reagents and competitive side reactions in complex physiological environments. For example, if the rate constant is 10 M^−1^ s^−1^ and the concentrations in vivo are both 10 μM, the half-life is supposed to be 2.8 h [48].

As a trigger of the click-to-release reaction, the stability of tetrazine under physiological conditions is also crucial to drug activation. Dimethyltetrazine **11** was reported to hydrolyze approximately 50% in phosphate-buffered saline (PBS) within 14 h and dipyridyl-tetrazine **12** with a shorter half-life of 9.6 h [56]. While some other alkyl- or pyridinyl-substituted tetrazines were relatively stable (maintained over 85% in PBS for 10 h at 37 °C) [73]. Therefore, it is critical to select the appropriate and stable tetrazine for the practical click-to-release system. Furthermore, as small molecules, dialkyl tetrazines are likely to clear from circulation too fast to be quantitatively reacted at the tumor site. Prolonging blood circulation time through proper structural modification without reducing reactivity or utilizing novel drug delivery systems to achieve targeted accumulation may become effective solutions.

On the other hand, TCO needs to have sufficient stability to ensure that it maintains enough reactivity after distribution and metabolism. However, previous studies determined that TCO can isomerize to the unreactive *cis*-isomer in the presence of thiols [74] and copper-containing serum proteins [75]. Within 7 h, the TCO almost completely converted into *cis* in 50% fresh mouse serum at 37 °C [75]. Finding ways to avoid contact can be helpful to prevent TCO from deactivation during circulation. Binding to an antibody with a short spacer, for example, TCO moiety was shielded by the antibody and stabilized with a half-life of 5 days in vivo [59]. Additionally, isomerization decreases reaction efficiency but has the merit of noninterference in payload release. Moreover, some highly reactive and stable *cis*-dioxolane-fused *trans*-cyclooctenes (d-TCO) derivatives have been developed recently [76], which would facilitate in vivo click-to-release efficiency in the future. However, synthesis of the TCO derivatives involves a complicated photochemical isomerization followed by tedious isolation of the axial isomer, leading to low overall yield [57,77]. Future work should address these deficiencies by developing more suitable synthetic pathways.

### 2.2. Tetrazine-Triggered Cleavage from Vinyl Ether

**Figure 4:**
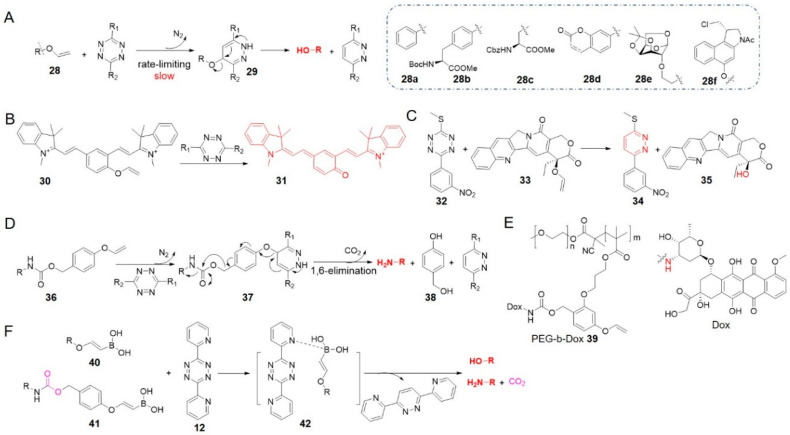
(**A**) Reaction mechanism between vinyl ether derivatives and tetrazines. (**B**) Structures of vinyl ether caged near-infrared fluorogenic probe and its active form. (**C**) The concept of prodrug-prodrug activation. (**D**) Reaction mechanism between vinyl ether self-immolative linker and tetrazines. (**E**) Structures of PEG-b-Dox and Dox. (**F**) Reaction mechanism between vinylboronic acids and tetrazines.

Other dienophiles have also been developed for tetrazine bioorthogonal cleavage reactions. In 2016 and 2017, the groups of Devaraj and Bernardes independently reported vinyl ether as a temporary mask which could be removed by reacting with tetrazine, releasing compounds containing free OH group (Figure 4A) [45,66]. IEDDA cycloaddition was the rate-limiting step, and after the expulsion of N_2_, aromatization of pyridazine drove swift release of the leaving group [66].

Devaraj et al. used tetrazine to activate a near-infrared cyanine dye caged by vinyl ether (**30** to **31** in Figure 4B) [45]. This chemistry can be combined with specific, sensitive reactions templated by nucleic acids for fluorescent RNA detection. Extending this reaction, Bernardes et al. installed a vinyl ether moiety on a range of hydroxyl-containing molecules, including amino acids **28b**, **28c**, a fluorophore **28d**, a monosaccharide **28e**, and an analogue of the cytotoxic drug duocarmycin **28f** (Figure 4A) [66]. Most of these vinyl ether derivatives remained intact over 8 h in vitro under conditions similar to physiological ones, and liberated alcohols in yields of 50–68% after reacting with tetrazine. It was demonstrated to be able to activate fluorescence of coumarin and release the drug duocarmycin in live cells. The universality of different chemotypes demonstrated the potential of this reaction for various chemical biology and medicine applications.

Interestingly, considering the formation of pyridazine product in tetrazine bioorthogonal reaction, Bradley et al. reported a mutual activation strategy termed as “prodrug-prodrug” activation (Figure 4C) [78]. In this strategy, tetrazine was also designed as a prodrug **32**, then reacted with vinyl ether-caged camptothecin **33** to generate the topoisomerase inhibitor camptothecin **35** along with a pyridazine-based microRNA 21 inhibitor **34**. Co-treatment of PC3 cells with 10 μM **32** and 0.5 μM **33** showed significant decrease in cell viability compared with the treatment of **33** alone (47 ± 8% vs. 101 ± 10%). Given that pyridazine is a scaffold in many drugs, the “prodrug-prodrug activation” strategy may be widely applicable. This approach may also support theranostics if one of the drugs is replaced with a diagnostic probe. In a similar manner, traceless Staudinger ligation and other bioorthogonal reactions can liberate phosphoramidate-containing prodrugs [27].

Besides hydroxyl group, amines can also be caged through a self-immolative linker and released by the click-to-release reaction between tetrazine and vinyl ether (Figure 4D) [79]. Bradley and his team designed a 4-hydroxymethyl phenyl vinyl ether linker, to which they conjugated fluorophores or drugs via a carbamate **36**. The 1,6-elimination followed by the liberation of phenolate **38** released amine compounds. Additional functionalization of the doxorubicin (Dox)-connected linker with a methacrylate led to the formation of amphiphilic PEG-b-Dox co-polymer nanoparticles **39**, which reacted with tetrazine to activate the cytotoxic Dox. Combining nanoparticles with tetrazine-based prodrug activation may offer new opportunities for targeted and controlled drug delivery.

Vinyl ethers are small, stable and easy to prepare, which may be useful for various applications in chemical biology and medicine. But the second-order rate constants (*k*_2_ = 3–5 × 10^−4^ M^−1^ s^−1^ in 10% H_2_O in DMF, entry 6 in Table 1) [66] are several orders of magnitude lower than those of TCO or other strained alkenes. Although vinyl ethers may function well in proximity-induced reactions such as those involving nucleic acids [80], the sluggish kinetics could be the major obstacle to its application in vivo [80].

Since vinylboronic acids were proven to be serviceable in bioorthogonal conjugation [81,82,83,84], Bonger’s group made an attempt to accelerate vinyl ether cycloaddition reactions by adding a boronic acid moiety on vinyl ethers (**40** in Figure 4F, entry 7 in Table 1) [67]. Using a tetrazine that bears boron-coordinating ligands, dipyridyl-tetrazine **12** for example, it was found that the reaction rate (*k*_2_ = 3.3 × 10^−3^ M^−1^ s^−1^ in 75% MeOH in PBS at 20 °C) was several times higher than that of vinyl ether [67]. Cycloaddition was followed immediately by the release of the alcohol or the amine via a self-immolative carbamate linker (**41**). When vinylboronic acid ether was employed to protect Dox, the prodrug reacted with tetrazine in HeLa cells, and the released drug inhibited cell growth efficiently [67].

### 2.3. Tetrazine-Triggered Cleavage from Benzonorbornadiene Derivative

The development of tetrazine-mediated removal of TCOs and vinyl ethers expanded the scope of bioorthogonal cleavage reactions, only partially met the requirements for in vivo applications. Exploring other reaction pairs with rapid reaction rate, near-quantitative release and prolonged serum stability is essential for more effective in vivo drug activation.

**Figure 5:**
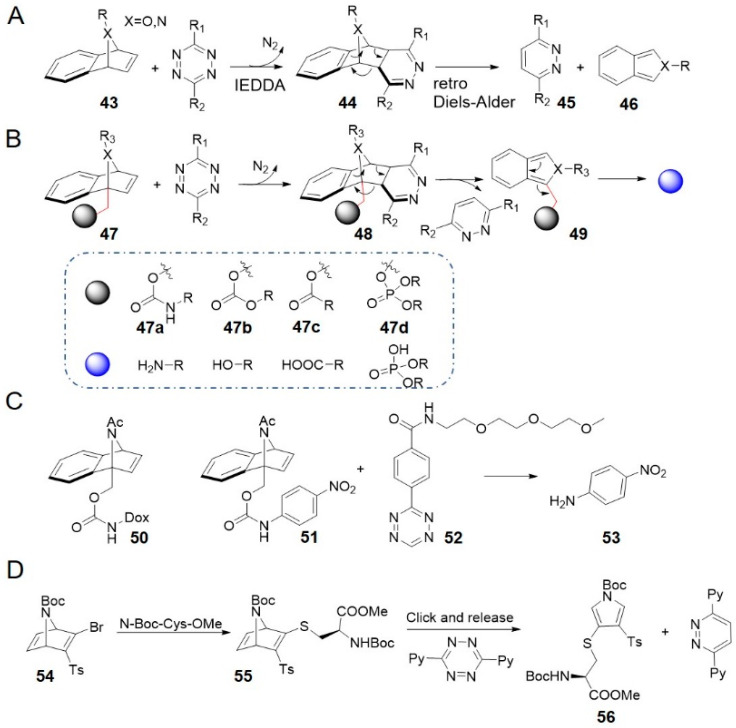
(**A**) Reaction mechanism between benzonorbornadienes and tetrazines. (**B**) Click-to-release reaction mechanism of tetrazine-triggered release from benzonorbornadiene derivatives and the range of corresponding leaving groups. (**C**) Examples of releasing *p*-nitroaniline and Dox. (**D**) Reactivity of thiol-conjugated azanorbornadiene towards tetrazines.

7-Aza/oxa-benzonorbornadienes **43** reacted with tetrazine through an IEDDA ligation to form dihydropyridazine adducts **44** which spontaneously underwent a retro Diels–Alder cycloreversion to aromatize and generate, isoindoles or isobenzofurans, respectively (Figure 5A) [85]. This reaction was used in tetrazine-mediated detection of microRNA [86]. What’s more, the reaction was developed into a bioorthogonal cleavage reaction by installing carbamate leaving groups to the bridgehead carbon of aza/oxa-benzonorbornadienes via a methylene linker (**47** in Figure 5B) [68]. Following similar mechanism, isoindoles or isobenzofurans **48** simultaneously release free amines due to the intrinsic lability. Benzonorbornadiene (BNBD) derivatives were highly stable under physiological conditions and reacted moderately with tetrazine to release cargo molecules almost quantitatively [68]. 7-Acetamide-BNBD **51**, for example, was stable in PBS-serum (1:1, *v/v*) for 7 days with no *p*-nitroaniline (compound **53**) or associated product observed. It reacted with PEG-tetrazine **52** at a rate of 0.017 M^−1^ s^−1^ (in 90% DMSO/H_2_O at 37 °C) and released approximately 90% of **53** (Figure 5C) [68]. Carbonates **47b**, esters **47c**, and phosphotriesters **47d** were released in as high yields as carbamates **47a** (Figure 5B) [87]. NAc-BNBDs showed higher release yields, while O-BNBDs reacted faster with tetrazine but the yields depended on reaction temperature. Reacting a Dox prodrug **50** with PEG-tetrazine **52** was proved to be as toxic as free Dox against A549 cells in culture (EC_50_ (**50**) = 96 nM vs. EC_50_ (Dox) = 88 nM) [68]. Stability of BNBD and facile drug release suggested higher potential for targeted drug delivery. Their reaction kinetics are faster than most bioorthogonal cleavage reactions, except for tetrazine click-to-release reactions with TCO and isocyanides (entries 8–10 in Table 1).

The long-term stability of benzonorbornadiene derivatives and their straightforward synthesis without the need for tedious stereoisomer separations make them promising for widespread applications in chemical biology. And the mutual orthogonality with other bioorthogonal release reactions allow to liberate two different drugs simultaneously [88]. However, the kinetics of benzonorbornadiene may need to be improved further before being used for prodrug activation at micromolar concentration in vivo.

Bernardes et al. have developed an azanorbornadiene bromovinyl sulfone reagent for cysteine-selective bioconjugation (Figure 5D) [89]. This reagent was demonstrated to be capable of reacting with the cysteine proteome in HeLa cell lysates. The most electron-rich double bond present in [2.2.1] bicyclic thiovinyl sulfone **55** underwent rapid IEDDA ligation with tetrazine **12** under aqueous conditions, followed by two consecutive *retro*-Diels-Alder reactions, generating a thiol-conjugated pyrrole **56** along with a pyridazine as a by-product. The initial cycloaddition step was rate-limiting, and the second-order rate constant was 0.026 ± 0.002 M^−1^ s^−1^ (at 37 °C), similar to that of aza/oxabenzonorbornadienes [68]. This bioorthogonal cleavage reaction may lead to new methods for stable, chemoselective bioconjugates.

### 2.4. Tetrazine-Triggered Cleavage from Isonitrile

**Figure 6:**
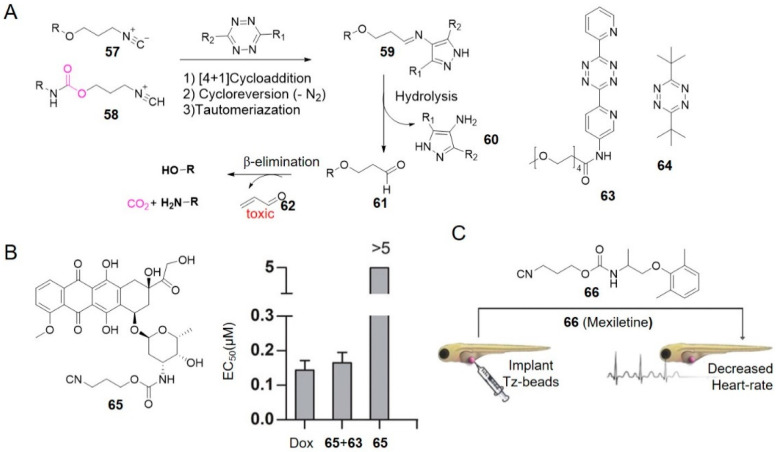
(**A**) Reaction mechanism between isonitriles and tetrazines. (**B**) Tetrazine-mediated activation of ICPrc-Dox prodrug. (**C**) Release of mexiletine in zebrafish embryos. Reproduced with permission from [70]; © 2020 American Chemical Society.

Isocyanides are another class of reagents that can undergo both bioorthogonal ligation [90] and cleavage reactions [70] with tetrazine. Franzini and co-workers used the removable 3-isocyanopropyl (ICPr, **57**) and 3-isocyanopropyl-1-carbamonyl (ICPrc, **58**) substituents as masking groups to chemically control the release of bioactive agents and probes (Figure 6A) [70,71]. **57** underwent a [4 + 1] cycloaddition with tetrazine followed by release of N_2_ and generation of an intermediate that tautomerized to a pyrazole-imine **59**. **59** spontaneously hydrolyzed to generate 4-aminopyrazoles **60** and 3-oxopropyl aldehydes **61**. Following the β-elimination reactions, **61** released acrolein **62** and hydroxy compound. Correspondingly, **58** linked with carbamates released amines [70].

ICPr **57** reacted with PEG-modified tetrazine **63** at a rate of 4.0 M^−1^ s^−1^ in PBS/DMSO (9:1) at 37 °C. With the catalysis of serum albumin, **57** could rapidly and almost quantitatively release the active molecules in PBS/serum (1:1), with a half-life of 166 s [70] (entries 11–12 in Table 1). Compared with dimethyl-tetrazine **11**, adding a bulky *tert*-butyl group on the tetrazine accelerated cycloaddition without destabilizing it [91]. *t*-Bu-tetrazine **64** for example, reacted with 2-phenylethyl isonitrile at a rate of 0.08 M^−1^ s^−1^ in DMSO/H_2_O (4:1) at 37 °C, which was 10.8-fold faster than **11** under the same condition. And no decomposition of **64** was observed in DMSO/H_2_O (4:1) containing 10 mM glutathione at 37 °C for 3 h. Since bulky groups always protect tetrazines from the approach of dienophiles [61,92], the opposite relation in reaction between bulky tetrazines and isonitriles suggested it may be orthogonal to strain-promoted azide-alkyne cycloaddition and IEDDA reaction between TCO and tetrazine, which was demonstrated by the triple-orthogonal labeling of proteins [91].

Authors also investigated the biocompatibility of this system in living cells and living organisms. High-yield drugs (91 ± 4%) were produced by reacting dipyridyl-tetrazine **12** (2 mM, 2 eq) with ICPrc-prodrugs of Dox **65** in PBS/DMSO (1:1, 37 °C). Combined treatment of excess **63** with **65** caused cell death in A549 adenocarcinoma cells efficiently (EC_50_ (**65**) = 0.165 ± 0.035 μM vs. EC_50_ (Dox) = 0.144 ± 0.028 μM, Figure 6B) [70], showing dose-dependent cytotoxicity. This strategy was validated again in vivo by implanting tetrazine-modified beads in zebrafish embryos and incubating with ICPrc-mexiletine **66**. The voltage-gated sodium channel blocker mexiletine was released leading to a decreased heart rate, which was consistent with in vitro results above (Figure 6C) [70].

Isocyanides are easy to synthesize and stable in biological fluids. In addition, ICPr and ICPrc are structurally compact, which makes them useful for creating prodrugs with minimal impact on the pharmacokinetics of parent drugs [70]. This innovative chemistry will create new opportunities for biomedical research and drug delivery. However, toxic acrolein as a by-product, may impose restrictions on in vivo applications. Further mechanistic study suggests the elimination reaction is hydrolysis-independent and may avoid the generation of acrolein [93].

### 2.5. Isonitrile-Triggered Cleavage from Tetrazine

**Figure 7:**
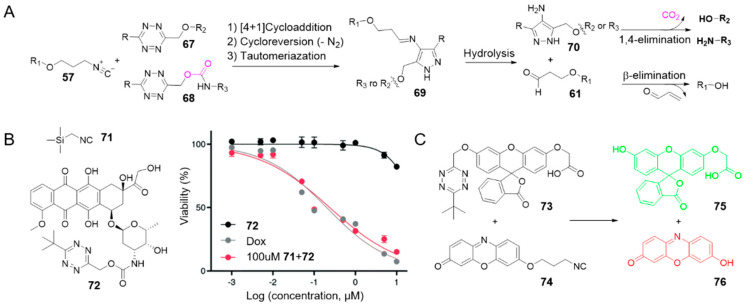
(**A**) Reaction between isonitriles and tetrazines leading to release of active molecules. (**B**) Structures of TMS-NC and Tzmoc-Dox prodrug, and statistical graph of cytotoxicity studies with A549 cells after 72 h. Reproduced with permission from [71]; © Royal Society of Chemistry. (**C**) Dual release of two fluorophores from ICPr- and TzMe-caged dyes.

Since the 4-aminopyrazole is a spontaneously eliminating functional group [94], the Franzini group reported the first example of release from tetrazine derivatives induced by isonitriles (Figure 7A) [71]. Following the similar mechanism, isonitriles converted tetrazines into 4-aminopyrazoles **70**. Through 1,4-elimination reaction of **70**, phenols and amines can be obtained from, tetrazylmethyl (TzMe) **67** and tetrazylmethyloxycarbonyl (Tzmoc) **68** derivatives respectively (Figure 7). The reaction of isonitrile-induced release is high-speed and near-quantitative in significant measure, especially with (trimethylsilyl)methyl isocyanide **71** (entries 13–14 in Table 1). Serum albumin can catalyze the elimination step. In addition, the compatibility of this chemistry with live systems was validated in vitro and in vivo. Upon reaction with **71** (100 μM), a prodrug of Dox (**72**) restored toxicity to A549 lung adenocarcinoma cells in vitro (EC_50_ (**72**) = 0.239 ± 0.014 μM vs. EC_50_ (Dox) = 0.202 ± 0.025 μM) (Figure 7B). By reacting TzMe-fluorescein **73** with ICPr-resorufin **74** in zebrafish embryos, double fluorescence turn-on could be simultaneously observed (Figure 7C) [71].

### 2.6. Cyclooctyne-Triggered Cleavage from Tetrazine

**Figure 8:**
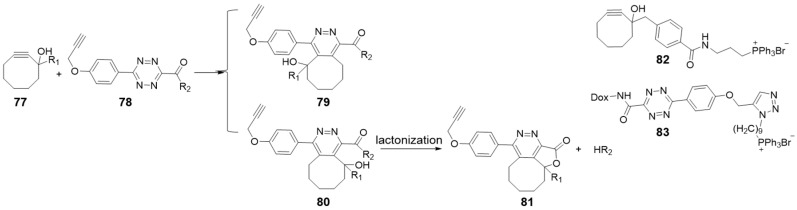
Reaction mechanism between cyclooctyne containing hydroxyl group and tetrazine. And structures of mitochondria-targeted cyclooctyne **82** and tetrazine-Dox prodrug **83**.

Chemists have gradually turned their attention to tetrazine as a prodrug linker in view of its two natural modification sites at 3 and 6 positions. Bioorthogonal cleavage can also be achieved by reacting cyclooctynes with tetrazines through a so-called “click, cyclize, and release” system (Figure 8) [36,95]. For instance, the cycloaddition reaction, between a cyclooctyne with a hydroxyl group at the propargyl position and a tetrazine with an amide-connected leaving group, led to lactonization with the active amine releasing [36,96]. The regiochemistry of the cycloaddition was that the nucleophilic hydroxyl group was positioned on the same side of the amide group (**80** vs. **79** in Figure 8), allowing effective lactonization. R_1_ groups can tune the second order rate constants in a range of 0.0075 to 0.25 M^−1^ s^−1^ and accelerate lactonization rate by adding conformational constrains (entry 15 in Table 1). The slow kinetics with a half-life longer than 100 h resulted in almost no possibility for the reaction pairs to click while circulating at low concentrations. However, they can be enriched at the specific area with a targeted modification (**82**, **83** in Figure 8). Then the locally increased concentrations can accelerate reaction rates and release drugs into targeted areas. Using this “click, cyclize, and release” approach, Wang et al. delivered and activated a Dox prodrug to mitochondria of HeLa cells in this kinetically controlled manner [36].

### 2.7. TCO-Triggered Cleavage from Tetrazine

**Figure 9:**
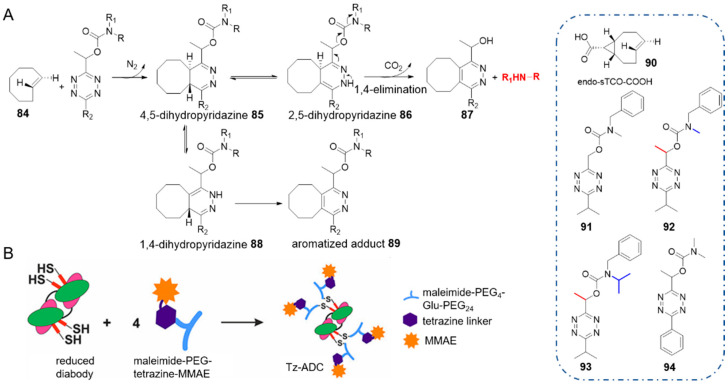
(**A**) Reaction mechanism between methylene carbamate-modified tetrazines and TCO. And structures of tetrazine and TCO derivates. (**B**) Schematic illustration of the Tz-ADC. Reproduced with permission from [65]; © 2020 American Chemical Society.

Robillard and co-workers reported a click-to-release strategy in which tetrazine carried the molecule of interest, and TCO served as the trigger for bioorthogonal activation [65]. Tetrazine bearing a methylene-linked carbamate reacted rapidly with TCO and a secondary amine was liberated. In this elimination, 4,5-dihydropyridazine **85** tautomerized to 2,5-dihydropyridazine **86**, which led to 1,4-elimination of the carbamate (Figure 9A). Installing a methyl substituent on the methylene facilitated cleavage (**92** vs. **91**), while the bulky *N*-isopropyl substituent stabilized the tetrazine carbamate (**93** vs. **92**).

As a result, these cleavable tetrazines maintained stable in serum or in the presence of thiols. Cycloaddition between TCO **84** and dimethylcarbamate-tetrazine **94** proceeded with a rate constant *k*_2_ of 3.14 ± 0.10 M^−1^ s^−1^ (at 20 °C in acetonitrile), which was 6-fold faster than cycloaddition between allyl-substituted TCO and 3,6-bisalkyltetrazine (entry 5 in Table 1). Using the more reactive sTCO-acid **90** led to a bimolecular rate constant up to 420 ± 49 M^−1^ s^−1^ in acetonitrile and 23,800 ± 400 M^−1^ s^−1^ in 25% MeCN/PBS [97] respectively. These rate constants are several orders of magnitude larger than that of using TCO as the caging molecule [56].

Then tetrazine was designed as a novel linker for antibody-drug conjugate (ADC). It connected the anti-tumor drug monomethyl auristatin E (MMAE) with a pegylated CC49 diabody which targets tumor associated glycoprotein 72 (TAG72), to form a Tz-ADC. Reaction between the Tz-ADC and TCO afforded 93% release of MMAE, which was greater than release yields of TCO-ADC [72]. The prodrug showed semblable cytotoxicity comparing with the parent drug against human colorectal cancer cells, with an EC_50_ value of 0.67 nM.

In this elimination approach, simpler TCOs that lack allylic substitutions, such as sTCO, can be used to trigger extremely fast reactions and therefore offer great possibilities for prodrug activation in vivo. In addition, acting as a linker, tetrazine maintained high reactivity and adequate stability, which conquered the defects of the primordial TCO-linker. Notwithstanding faster cycloaddition and near-quantitative release were offered, the scope for functional groups of this elimination strategy should be expanded in further research.

## 3. In Vivo Applications of Prodrug Delivery and Activation

For in vivo use, cleavage chemistry should provide high spatiotemporal resolution as well as chemoselectivity. Numerous first-line drugs, such as antitumor drugs, often cause severe toxic effects and show poor efficacy [98]. As an alternative, antitumor drugs could be delivered to tumor sites taking advantage of novel drug delivery systems and activated specifically through tetrazine-responsive bioorthogonal cleavage chemistry, which may reduce the toxicity and improve the efficacy. Consistent with this notion, recent investigations on optimized prodrug delivery and release in vivo are summarized below.

### 3.1. Targeted Drug Delivery Based on Hydrogels

**Figure 10:**
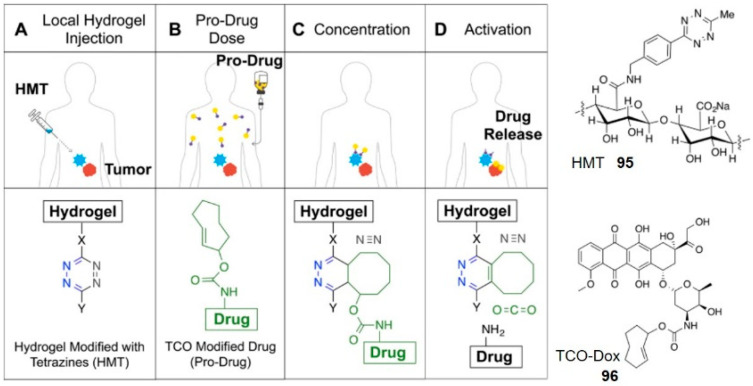
Schematic illustration of the local drug activation approach: a hydrogel comprising alginate monosaccharides modified with tetrazine was co-administered with TCO-Dox. The prodrug conjugate accumulated in the hydrogel where it was also activated by the tetrazine. Reproduced with permission from [100]; © 2020 American Chemical Society.

Hydrogels are cross-linked, three-dimensional, macromolecular hydrophilic networks. The high-water content and soft consistency of hydrogels with the similar features of natural extracellular matrix make them suitable for drug delivery [99]. The advance of click-to-release chemistry enables tetrazine to locally trigger and activate the prodrug on delivery. For example, the alginate hydrogel decorated with tetrazine moieties **95** was preinjected into soft tissue sarcoma xenografts in mice through palpation, and TCO-Dox **96** was injected intravenously subsequently (Figure 10) [100]. The hydrogel concentrated and activated the prodrug at the tumor site, leading to greater antitumor efficacy and lower myelosuppression than free Dox control. In another example, Czuban and co-workers developed a reloadable hydrogel platform for antibiotic prodrugs, which was able to inhibit the planktonic and biofilm growth of bacteria efficiently [101]. Injectable hydrogel-based activation may be particularly well suited to cancer therapy because cytotoxic drugs can be concentrated and locally activated at the tumor area. This will allow giving larger therapeutic doses under a better safety profile. Gratifyingly, a first-in-human Phase I clinical trial has been approved by the FDA recently [47]. It’s worth looking forward to whether this new research will bring new opportunities and challenges.

### 3.2. Targeted Drug Delivery Based on Nano Transporters

Prodrugs, like the parent drugs, may suffer from nonspecific distribution, poor water solubility, and low persistence in circulation. Encapsulating prodrugs into nanoparticles can enhance the stability and modulate the biodistribution of the drugs. These compounds are able to reduce systemic toxicity by targeting specific tumor sites [102]. Nanovehicles can also be functionalized in different ways as versatile therapeutic tools. Several nano-platforms based on bioorthogonal click-to-release chemistry have been developed to improve the efficiency of prodrug delivery and activation.

**Figure 11:**
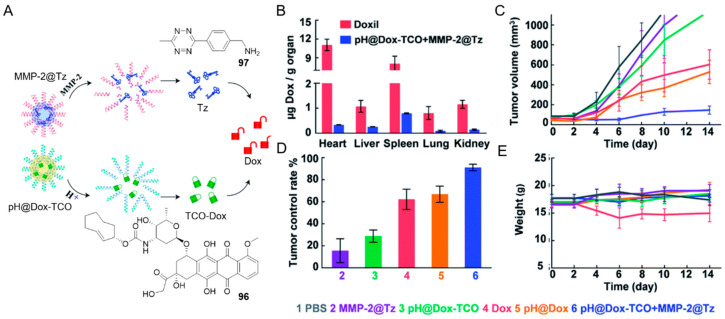
(**A**) Schematic illustration of co-administration of two nanovehicles responsive to the tumor microenvironment. (**B**) Concentrations of Dox in main organs after two-hour injection. (**C**) Tumor volumes, (**D**) Tumor control rate and (**E**) Body weight of mice under different conditions at different time points. Reproduced with permission from [103]; © Royal Society of Chemistry.

For example, the TCO-Dox and tetrazine were packaged separately into micelles and were designed to be dissociated in the presence of matrix metalloproteinase 2 at low pH, which is a characteristic condition of the tumor microenvironment (Figure 11A) [103]. 

When both TCO-Dox and tetrazine **97** were present in this microenvironment, activation of the prodrug shall be triggered. This hierarchically regulated strategy inhibited tumor development in 4T1 tumor-bearing mice effectively (Figure 11C–E). This also led to approximately 32.6 times reduction in biodistribution to heart when comparing to Doxorubicin Hydrochloride Liposomal (Doxil) after two-hour systemic administration (Figure 11B). Since cardiotoxicity is one of the main side effects of Dox, this approach with lower toxicity may pave the way to new effective cancer therapy.

**Figure 12:**
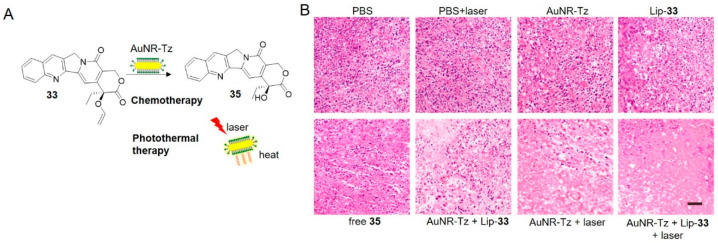
(**A**) Schematic illustration of gold nanorods supported chemotherapy and photothermal therapy. (**B**) H & E staining of tumor biopsies from different treatment groups. Reproduced with permission from [106]; © 2020 American Chemical Society.

Gold nanorods (AuNRs) are another desirable vehicle for combination therapy due to contribution in photothermal therapy [104,105]. It was convinced that the combination of chemo- and photothermal therapy inhibited tumor growth (Figure 12) [106]. The AuNR-Tz trigger, prepared by fixing PEGylated tetrazine onto AuNRs, can convert light to heat and are therefore useful for photothermal therapy as well as optoacoustic imaging. Camptothecin was masked by vinyl ether (prodrug **33**) and encapsulated into liposomes. Upon intravenously injected to HeLa-tumor-bearing mice, the two components accumulated at the site of tumor solely, as revealed by fluorescence imaging and multispectral optoacoustic tomography imaging. Tetrazine on the gold nanorods activated the prodrug, while the combination of chemo- and photothermal therapy inhibited tumor growth effectively, resulting in extremely low relative tumor volume and very high apoptosis level among all treatment groups (Figure 12B).

### 3.3. Theranostics

Systems that can deliver drugs to disease sites while simultaneously allowing those sites to be imaged make it much easier to examine where drugs are acting in the body and how the body responds to the treatment [107]. In addition, the drug can be administered more selectively under imaging guidance. Several studies have substantiated the crucial role of nanomaterials for diagnosis and imaging based on tetrazine-responsive bioorthogonal cleavage [108,109,110,111]. For example, a vinyl ether-caged camptothecin prodrug and tetrazine-quenched near-infrared fluorophore were separately encapsulated into liposomes, which localized to tumors due to the EPR effect [112]. Reaction between tetrazine and vinyl ether in situ turned on fluorescence and inhibited tumor growth.

**Figure 13:**
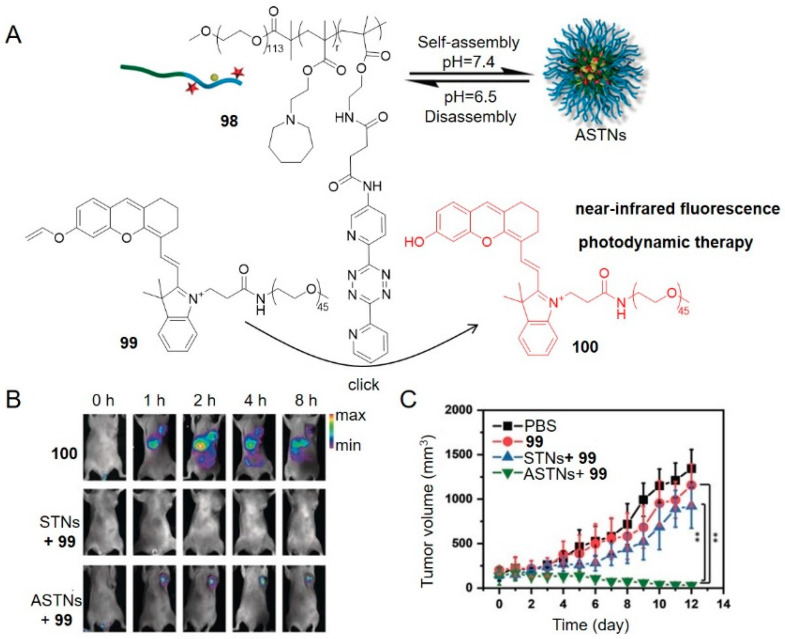
(**A**) Schematic illustration of activation of vinyl ether-caged fluorogenic probe (CyPVE) by a pH-responsive tetrazine polymer. (**B**) In vivo fluorescence imaging at different time after injection. (**C**) Tumor growth curves. ASTNs, pH-sensitive nanoparticles; STNs, pH-insensitive nanoparticles. Reproduced with permission from [87]; © 2020 Wiley-VCH Verlag GmbH & Co. KGaA, Weinheim.

In another case, a tetrazine-modified polymer **98** disassembled at pH 6.5 in the tumor microenvironment (Figure 13) [87]. This disassembly process allowed tetrazine to activate a vinyl ether-caged near-infrared hemicyanine dye **99**, which fluoresced and generated singlet oxygen, causing tumor cell death through the photodynamic effect. Tetrazine polymers were first intravenously injected to 4T1 breast tumor-bearing mice followed by injection of **99** two hours later, and lastly tumors were exposed to 660 nm laser (0.20 W cm^−2^) for 20 min. The significant fluorescence and growth inhibition of tumor tissues were observed in treatment group (Figure 13B,C).

### 3.4. Enzyme-Instructed Supramolecular Self-Assembly (EISA)

**Figure 14:**
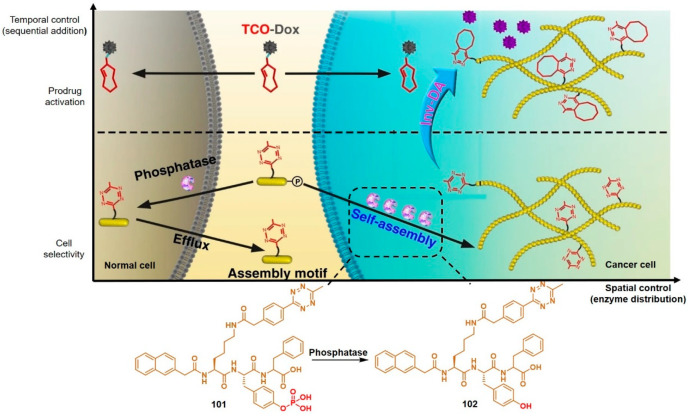
Synergistic enzymatic and bioorthogonal reaction for prodrug activation in tumor cells. Reproduced with permission from [98]; © 2020, Springer Nature.

Taking advantage of the initiation of the supramolecular self-assembly of small molecules by upregulated phosphatase level in tumor cells, the research group of Chen linked tetrazine to a self-assembly tri-peptide motif KYF to build the prodrug activation trigger **101** (Figure 14) [98]. 

The phosphorylated tyrosine responded to overexpressed phosphatase, resulting in so-called enzyme-instructed supramolecular self-assembly (EISA) which specifically accumulated tetrazine in cancer cells. TCO-Dox (30 mg kg^−1^) was intravenously injected in HeLa tumor-bearing mice two hours after the administration of **101** (50 mg kg^−1^). Activation of TCO-Dox led to greater cytotoxicity than the control group without EISA. This spatiotemporally controlled prodrug activation may serve as a small-molecule strategy for mitigating the adverse effects of chemotherapy.

### 3.5. Antibody-Drug Conjugates (ADCs)

Drugs can target tumor cells with high selectivity and affinity by conjugating to antibodies targeting specific antigens, which terms as antibody-drug conjugates (ADCs) [113]. Classic activation of ADCs includes several processes: (1) binding to target antigen on the tumor cell surface, (2) internalization by receptor endocytosis into tumor cell, (3) cleavage of the linker and release of an active form of cytotoxic moiety of ADCs [114]. However, inefficient internalization limits ADCs applications, especially in solid tumors. In contrast, chemically triggered activation through bioorthogonal cleavage reaction gives manifold possibilities to deliver drugs actively via internalized receptors, non-internalized receptors, cytoplasmic proteins and even extracellular matrix components.

**Figure 15:**
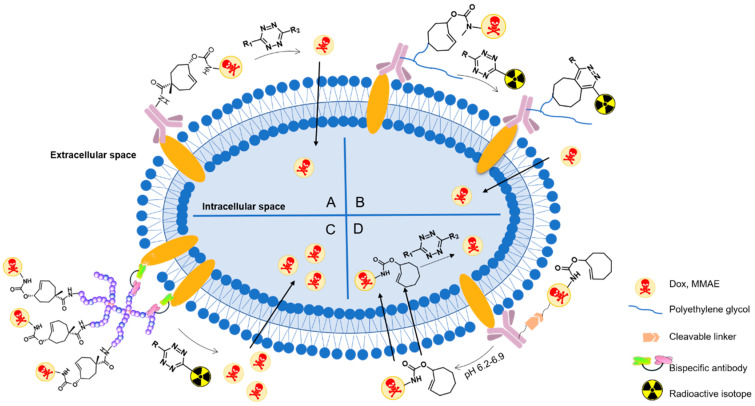
“Click-to-release” activation of an antibody-drug conjugate. (**A**) First demonstration of an ADC in vivo. (**B**) Chemically triggered drug release from a diabody-based ADC. (**C**) Hyperbranched polymer-based bioorthogonal approach for drug delivery. (**D**) Prodrug-antibody conjugates for targeted chemotherapy. ADC, antibody-drug conjugate; Dox, doxorubicin; MMAE, monomethyl auristatin E.

**Figure 16:**
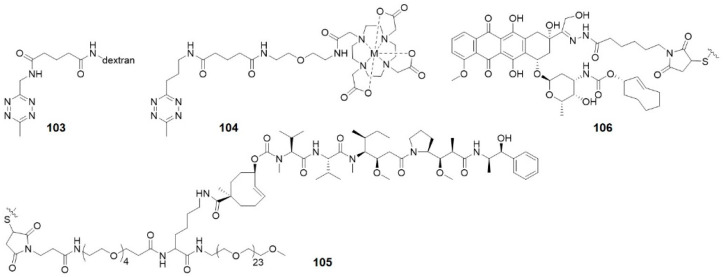
Structures of compounds used in ADCs studies.

Robillard and co-workers were the first to validate the potential of click-to-release reaction between TCO and tetrazine for ADC activation [59]. They linked TCO-Dox prodrug to a monoclonal antibody (mAb CC49) against tumor-associated glycoprotein-72, which is a non-internalizing cancer target (Figure 15A). This model ADC showed high tumor uptake of 30–40% ID/g at 30 h after injection (5 mg kg^−1^) to LS147T tumor-bearing mice and could be selectively activated by tetrazine. However, release yields of Dox were not satisfactory. This is most probably the result of rapid clearance of tetrazine. Dimethyl-tetrazine **11**, for example, was not able to activate TCO-Dox significantly. Whereas with a modification of dextran, **103** (Figure 16) gave 51.3% release of Dox at 24 h after injection. Therefore, the choice of tetrazine that persists longer in circulation is crucial.

The same research group improved the ADC approach by using PEGylated diabody conjugates instead of intact antibodies, reducing off-target effect of the ADCs (Figure 15B) [72]. PEG_24_ residues were conjugated to CC49, leading to high tumor uptake and fast blood clearance. The tubulin-binding antimitotic monomethyl auristatin E (MMAE), a classic cytotoxic drug for ADCs, was caged by TCO to form the prodrug. Then the prodrug was linked to PEG residues via a lysine-branched spacer to establish linker-drug building block **105**. Tetrazine **104** was delivered as a conjugate with tetraazacyclododecane-1,4,7,10-tetraacetic acid (DOTA), which reduced blood clearance rate to allow more complete drug to enrich, and also enabled imaging of the ADC via single-photon emission computed tomography (SPECT). This may provide potential applications in theranostics. Combination of ADC and **104** showed strong antitumor activity in LS174T and OVCAR-3-tumor-bearing mice without observed toxicity.

The toolbox of TCO moieties that can be directly attached to an antibody without interfering its bio-function is to be expanded. Thurecht’s group developed a novel polymer-drug conjugate for a three-component system (Figure 15C) [115]: (1) a TCO-Dox prodrug conjugated to a PEGylated hyperbranched polymer (HBP); (2) a PEGylated tetrazine trigger functionalized with the ^64^Cu chelator NOTA; (3) a bispecific antibody (BsAb) that recognized tumor-associated glycoprotein-72 as well as PEGylated nanoparticles via strong non-covalent interactions. Firstly, the HBP-TCO-Dox was incubated with BsAb (at a ratio of 1:1) for one hour and then the conjugate was injected intravenously to MCF7-tumor-bearing mice. 24 h later, [^64^Cu] tetrazine-PEG_4_-NOTA was injected. The click-to-release reaction released Dox into cancer cells, and the Dox could be quantitated based on ^64^Cu radioactivity using PET-CT. This system may have several advantages over other ADCs systems: PEGylated HBPs prolonged the circulation time, reduced immunogenicity, and facilitated theranostics. The possibility of real-time imaging can support detailed studies of pharmacokinetics and pharmacodynamics, which can in turn improve future development of nanotherapeutics. This system may be adaptable to treatments of various tumor types by using bispecifics that contain two antibodies targeting different antigens and Fc region creating a third functional binding site to facilitate antibody dependent cell-mediated cytotoxicity (ADCC).

ADC-delivered drugs that act on intracellular targets need to diffuse into the target cells, which may be blocked by protonation. One solution is to firstly release a more permeable prodrug instead of the active drug. Lately, the prodrug can be activated by small molecule tetrazine activators. For example, TCO-Dox (**106**) was linked to Her-2 antibody via a pH-sensitive linker. At low pH, this linker would be cleaved and TCO-Dox was released extracellularly and activated within tumor cells (Figure 15D) [116]. This ADC was intravenously injected in MDA-MB-231-tumor-bearing mice and exerted cytotoxicity after the administration of tetrazines. This combination strategy improves liberation of Dox inside tumors and reduces Dox-related toxicity. However, there is still a risk of off-target toxicity in response to tumor microenvironment since the prodrugs are not completely non-toxic.

In this case, non-internalizing antigens can be selected as targets, which expands the range of possible target-antigen selections. In addition, further study on enhancing the stability and tumor uptake of ADCs as well as improving the activity and residence time of activators is needed.

## 4. Summary and Outlook

With the understanding of the tetrazine bioorthogonal chemical reaction progress, through rational designs of chemical structures, most of bioorthogonal reaction partners can occur either a ligation or a cleavage reaction with tetrazine. Tetrazines undergo cycloaddition reactions with various reactants, followed by elimination or rearrangement to cleave specific chemical bonds and release active molecules. In these researches discussed above, the stability of substrates, reaction kinetics, release yields and the scope of releasable functional groups have been thoroughly explored and carefully investigated. These diverse reactions can release drugs bearing diversiform functional groups, including amino, hydroxyl and carboxyl groups. Future studies should explore the possibility of liberating other active functional groups, such as sulfonic and phosphoric acids.

Due to lower toxicity, prodrugs allow larger administration dosage by comparison to parent drugs and therefore contribute to higher efficacy after activation. Moreover, the employment of exogenous trigger containing a reporter like fluorophore or radioactive isotope for drug activation can simultaneously provide imaging signals to achieve diagnosis and treatment. In addition, recent examples about conditional activation with multiple controls, such as singlet oxygen and light, are expected to provide improved spatial and temporal resolution [117,118]. Furthermore, there are still many challenges in establishing bioorthogonal platforms for multi-drug release and clinical applications in theranostics [119], which will likely require further developments in bioorthogonal chemistry and biomaterials.

Improving reaction kinetics and the stability of reactants is also a prerequisite for in vivo applications. This will ensure that the two reactants can still reach effective concentrations at the target site after circulation and distribution so that the reaction can be carried out on a reasonable timescale. Additionally, it is worth noting that giving two molecules rather than one requires stricter safety regulations and both of the two need to meet the FDA approval for clinical treatment. The possibility and effectiveness of clinical transformation are under study. It is a long-term and formidable process from the success of animal experiments to the final clinical use, but we still believe future chemical biology advancements will spur the process onward. Other questions including whether other routes of administration, such as oral delivery, are feasible and whether relevant reagents would survive first pass through the liver and the P450 enzymes, are worthy of further exploration to promote these methods to meet clinic needs imminently.

In current tetrazine-responsive bioorthogonal prodrug activation strategies, both tetrazines and dienophiles have the potential to be employed as triggers or molecule cages. Moreover, two natural modifiable positions of the tetrazine molecule make it convenient to modify with functional handles. With the development of tetrazine synthesis methodology, the employment of tetrazine as a cage for proteins, fluorophores, etc., may become a hot topic of research. It is convinced that more chemical biology tools will be developed soon to meet the clinical needs.

## Figures and Tables

**Table 1:** Summary of tetrazine-responsive bioorthogonal cleavage reactions.

	Pro-Moiety	Trigger	*k*_2_ (M^−1^ s^−1^)	Release Kinetics	Yield of Release (%)	Ref
1	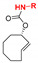	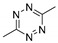 t_1/2_ = 14.4 h ^1^	48.7 ± 1.1	complete < 30 min	79	[56]
2	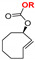		8.5 ± 0.7	t_1/2_ < 3 min	89	[57]
3	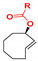		15.2 ± 0.5	t_1/2_ < 3 min	90	[57]
4	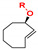		9.4 ± 0.2	t_1/2_ < 10 min	80~90	[57]
5	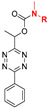	** 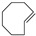 **	287 ± 10	t_1/2_ = 10 h	80	[65]
6	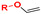	** 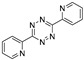 **	3.92 × 10^−4^	NA ^2^	61	[66]
7	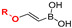	** 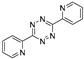 **	3.3 × 10^−3^	NA ^2^	<80	[67]
8	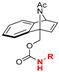	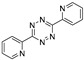 t_1/2_ = 9.6 h ^1^	0.02	NA ^2^	87.5	[68]
9	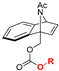		0.017	NA ^2^	94.7	[69]
10	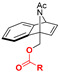		0.027	NA ^2^	96	[69]
11	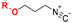	** 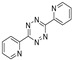 **	4.0	*k_elim_* = 1.6 × 10^−4^ s^−1^	99.1	[70]
12	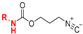		1.1	*k_elim_* = 5 × 10^−5^ s^−1^	93.5	[70]
13	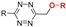	** 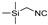 **	0.301 ± 0.018	*k_elim_* = 3.5 × 10^−3^ s^−1^	100	[71]
14	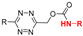		0.344 ± 0.013	*k_elim_* = 3.4 × 10^−4^ s^−1^	100	[71]
15	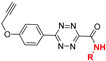	** 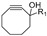 **	0.25	*k_elim_* = 8.05 × 10^−6^ s^−1^	80~90	[36]

^1^ Stability assessed in PBS at 37 °C [56]. ^2^ Elimination kinetics is not assessed since the first step is the rate-limiting step. NA stands for not assessed.
